# Differential identity of Filopodia and Tunneling Nanotubes revealed by the opposite functions of actin regulatory complexes

**DOI:** 10.1038/srep39632

**Published:** 2016-12-23

**Authors:** Elise Delage, Diégo Cordero Cervantes, Esthel Pénard, Christine Schmitt, Sylvie Syan, Andrea Disanza, Giorgio Scita, Chiara Zurzolo

**Affiliations:** 1 Unité Trafic Membranaire et Pathogenèse, Institut Pasteur, 25–28 Rue du Docteur Roux, 75724 Paris CEDEX 15, France; 2 Ultrapole, Institut Pasteur, 25-28 Rue du Docteur Roux, 75724 Paris CEDEX 15, France; 3 FIRC Institute of Molecular Oncology, 20139 Milan, Italy; 4 Dipartimento di Scienze della Salute, Università degli Studi di Milano, 20122 Milan, Italy

## Abstract

Tunneling Nanotubes (TNTs) are actin enriched filopodia-like protrusions that play a pivotal role in long-range intercellular communication. Different pathogens use TNT-like structures as “freeways” to propagate across cells. TNTs are also implicated in cancer and neurodegenerative diseases, making them promising therapeutic targets. Understanding the mechanism of their formation, and their relation with filopodia is of fundamental importance to uncover their physiological function, particularly since filopodia, differently from TNTs, are not able to mediate transfer of cargo between distant cells. Here we studied different regulatory complexes of actin, which play a role in the formation of both these structures. We demonstrate that the filopodia-promoting CDC42/IRSp53/VASP network negatively regulates TNT formation and impairs TNT-mediated intercellular vesicle transfer. Conversely, elevation of Eps8, an actin regulatory protein that inhibits the extension of filopodia in neurons, increases TNT formation. Notably, Eps8-mediated TNT induction requires Eps8 bundling but not its capping activity. Thus, despite their structural similarities, filopodia and TNTs form through distinct molecular mechanisms. Our results further suggest that a switch in the molecular composition in common actin regulatory complexes is critical in driving the formation of either type of membrane protrusion.

Tunneling Nanotubes (TNTs) are cellular protrusions that represent a mechanism for direct, long-range intercellular communication^1^. They constitute a membranous and cytoplasmic continuity between remote cells supported by the actin cytoskeleton and in some cases microtubules^1^,^2^. TNTs are fragile and dynamic structures with a small diameter (20–500 nm) and a length up to 100 μm, which hover freely in the medium without touching the substrate in culture. They have been shown to mediate the cell-to-cell transfer of many different cellular components including: membrane proteins, soluble molecules, vesicles derived from various organelles, and mitochondria^3^. TNT-like structures have been observed in a wide variety of cell types *in vitro*^2^ as well as *in vivo* models^4^,^5^,^6^,^7^. Even though the precise physiological function of TNTs remains enigmatic, their involvement in essential processes like signal transduction, apoptosis, development, and immune response has been postulated^2^,^8^. Various pathogens, such as viruses^9^,^10^ and bacteria^11^, can use TNT-like structures to travel from one cell to another. TNTs are also emerging as an important player in cancer development^5^,^6^,^12^,^13^. We have previously demonstrated that TNTs can mediate the intercellular transfer of infectious prions between neuronal cells, dendritic cells to neurons, and between astrocytes^14^,^15^,^16^,^17^. Interestingly, other “prion-like” amyloidogenic proteins like misfolded huntingtin^18^, amyloid β^19^, α-synuclein^20^, and tau^21^ can also be transferred between distant cells through TNTs, thus underscoring the important role of TNTs in the progression of neurodegenerative diseases, and their potential use as therapeutic targets^22^.

Two mechanisms for TNT formation have been proposed^23^. The first one, which is commonly referred to as the “cell dislodgment mechanism”, describes two cells closely apposed to each other fusing transiently and subsequently retaining a thin thread of membrane while they move apart^11^. An alternative mechanism, known as the actin driven protrusion mechanism, proposes an active process based on the extension of a filopodium-like protrusion from one cell to another, followed by membrane fusion of the tip upon physical contact^1^. In both cases, the application of actin depolymerizing drugs strongly reduces TNT formation, suggesting that actin plays a critical role^1^,^24^,^25^. However the molecular mechanism(s) underlying TNT formation is still ill defined. The role of endogenously and exogenously expressed M-Sec, a protein sharing homology with Sec6, a component of the exocyst complex, as a positive regulator of TNT formation has been shown in multiple cell types^9^,^26^,^28^. M-Sec induction of TNT formation involves its interaction with the GTPase Ras-related A protein (RalA) and the exocyst complex^26^. Furthermore, the transmembrane major histocompatibility complex (MHC) class III protein leucocyte specific transcript 1 (LST1) interacts with M-Sec and mediates the recruitment of RalA to the plasma membrane, promoting its interaction with the exocyst complex^27^. This multi-molecular complex can contribute to the remodeling of the actin cytoskeleton and to the delivery of membrane at the site of TNT formation. The protein p53 was recently found to play a crucial role in the formation of TNTs in astrocytes via the epidermal growth factor and the Akt/mammalian target of rapamycin (mTor)/phosphatidylinositol 3-kinase (PI3K) pathway^19^. However, cells that do not express M-Sec, such as the mouse neuronal CAD cell line and neurons, are still capable of forming TNTs^29^. Furthermore, p53-independent TNT formation was observed in rat pheochromocytoma PC12 cells and in acute myeloid leukemia cells^29^, suggesting that different molecular mechanisms may be at play. Whether different formation mechanisms lead to intercellular connections having distinct functions remains to be determined^30^.

Another important observation is that TNTs share structural similarities with filopodia, particularly in their small diameter and requirement of actin for the protrusion^31^. We have previously observed that the expression of two known filopodia inducers, the vasodilatator-stimulated phosphoprotein (VASP) and fascin^32^, decreases the number of TNT-connected cells^30^ while Myosin X, another filopodial inducer, stimulates the formation of TNTs and intercellular transfer of vesicles in CAD cells^33^. We further demonstrated that TNT induction requires the F2 lobe of Myosin X “band 4.1, ezrin, radixin, moesin” (FERM) domain^30^, but not the F3 lobe (both needed for filopodia adherence to the substrate^34^,^35^, suggesting that dorsal filopodia might be a TNT precursor. However, these findings and other data in the literature did not reveal whether TNTs and filopodia are identical or structurally related structures, and whether they share the same machinery for their formation.

To address this issue we studied the role of a subset of different actin remodeling complexes in TNT formation previously shown to be involved in filopodial protrusion. Specifically, up-regulation of filopodia elongation was shown to depend on VASP recruitment and clustering by the Insulin Receptor Substrate of 53 kDa (IRSp53)^36^. IRSp53 has been implicated in the formation of filopodia via its dual function: it can deform the plasma membrane through its “Inverted Bin-Amphiphysin-Rvs” (I-BAR) domain, thus generating a membrane protrusion, and it interacts with several actin regulators via its “Src homology 3” (SH3) domain^37^,^38^. IRSp53 is synergistically activated by the binding of effectors to its SH3 domain and by the binding of the RhoGTPase CDC42 to its “CDC42 and Rac interactive binding” (CRIB) domain^39^. Thus, we investigated the possible role of IRSp53 and CDC42 in the formation of TNTs and intercellular transfer of vesicles. We found that CDC42, IRSp53, and VASP act as negative regulators of TNT formation and vesicle transfer function. We further show that these proteins act in concert to inhibit TNT formation. We also demonstrated that epidermal growth factor receptor pathway 8 (Eps8), another actin regulator that has been shown to inhibit filopodia formation in neurons^38^,^40^, is a positive regulator of TNT formation and transfer. In addition, by expressing mutant forms of Eps8 which were impaired in either the capping or bundling activity of the protein, we showed that TNT induction requires Eps8 bundling but not capping activity. To our knowledge, this is the first molecular demonstration that TNTs and filopodia are different cellular structures that form through different mechanisms, paving the way to further study their physiological function(s).

## Results

### CDC42, IRSp53, and VASP negatively regulate TNT formation

**Figure 1:**
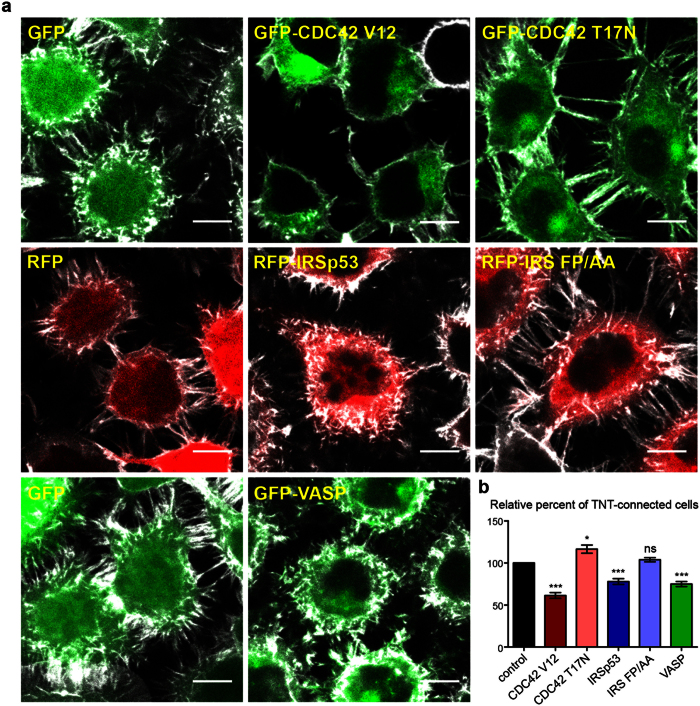
VASP, CDC42, and IRSp53 negatively regulate the number of TNT-connected cells. (**a**) Representative confocal images showing intercellular connections upon ectopic expression of GFP-CDC42 V12 (constitutively active form), GFP-CDC42 T17N (dominant negative form), RFP-IRSp53, RFP-IRS FP/AA (an IRSp53 SH3-mutant defective in its binding to ligands), or GFP-VASP. Cells were fixed 14 hrs post-transfection, labeled with WGA-Alexa Fluor^©^-647 nm (grey) in order to detect TNTs and observed by confocal microscopy. Scale bar = 10 μM. (**b**) Quantification of TNT-connected cells upon ectopic expression of GFP-CDC42 V12, GFP-CDC42 T17N, RFP-IRSp53, RFP-IRS FP/AA, or GFP-VASP. The ratio of TNT-forming transfected cells/number of transfected cells was evaluated. Data represent the mean (±SEM), normalized to control cells (GFP or RFP transfected cells) arbitrarily set at 100%, of at least 6 independent experiments. *P < 0.05; **P < 0.01; ***P < 0.001; ns = not significant.

In order to understand the relationship between filopodia and TNTs, and to dissect the molecular players involved in TNT generation, we investigated the role of actin regulators known to be involved in filopodia formation. We first transfected neuronal CAD cells^18^,^20^,^30^ with plasmids encoding fluorescently-tagged versions of these proteins and evaluated the ratio of TNT-connected cells in sub-confluent cultures by confocal microscopy. Around 65% of the control cells transfected with an empty vector encoding a fluorescent tag were connected by TNTs. Interestingly, the ectopic expression of GFP-CDC42 V12, a constitutively active form of CDC42, reduced the number of TNT-connected cells by approximately 40%, while GFP-CDC42 T17N, a dominant-negative form of the protein, significantly increased TNT formation (Fig. 1a and b, Supplementary Fig. 1). This suggests that, contrary to what has been reported in other cell lines^26^,^27^,^41^, CDC42 may negatively regulate TNT formation in neuronal CAD cells.

A significant reduction of TNT-connected cells was also observed upon ectopic expression of RFP-IRSp53 while expression of RFP-IRS-FP/AA, an IRSp53 mutant impaired in its binding to downstream effectors through the SH3 domain, did not affect TNT formation (Fig. 1a and b, Supplementary Fig. 1). This result points to the potential role of IRSp53 in the negative regulation of TNTs via its interaction with protein partners. Consistently, ectopic expression of GFP-VASP, an interactor of IRSp53, also leads to less cells connected via TNTs compared to the control (Fig. 1a and b, Supplementary Fig. 1).

The Ras-related C3 botulinum toxin substrate 1 (Rac1), a member of the ras-related superfamily of small GTPases has been shown to share the same signaling pathway with and be activated by CDC42^42^. To test if Rac1 has a similar effect on TNT formation as CDC42 in CADs, we tested Myc-Rac1 V12, a constitutively active form of Rac1, and Myc-Rac1 17 N, a dominant negative form of Rac1. We evaluated any changes in TNT formation and discovered that the active form of Rac1 decreases the percentage of cells connected via TNTs while its dominant negative form increases TNT formation, similar to the effect of the active and negative forms of CDC42, respectively (Supplementary Fig. 2). Thus, the combined use of Rac1 and CDC42 mutants provide evidence that inducers of filopodia and lamellipodia have an opposite effect (i.e. decrease) on TNT formation.

### CDC42, IRSp53, and VASP negatively regulate intercellular vesicle transfer

**Figure 2:**
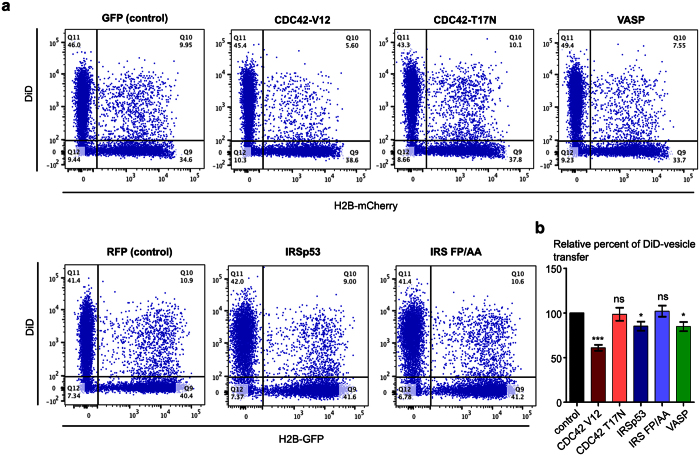
CDC42, IRSp53, and VASP negatively regulate intercellular vesicle transfer. (**a**) Raw data (dot plots) from a representative experiment showing the transfer of DiD-labeled vesicles to the acceptor population (H2B-mCherry) upon ectopic expression of GFP-CDC42 V12, GFP-CDC42 T17N, RFP-IRSp53, RFP-IRS FP/AA, or GFP-VASP in the donor population. A population of CAD cells was transiently transfected with the different fluorescently-tagged constructs (donor population) and another population was transiently transfected with either H2B-GFP or H2B-mCherry (acceptor population). Internal vesicles of donor cells were labeled with the membrane dye Vybrant DiD as described in the ‘Material and Methods’ section. Donor and acceptor cells were co-cultured for 16 hrs. Cells were then fixed and analyzed by flow cytometry. (**b**) Quantification by flow cytometry of DiD-positive acceptor cells upon ectopic expression of GFP-CDC42 V12, GFP-CDC42 T17N, RFP-IRSp53, RFP-IRS FP/AA, or GFP-VASP in the donor population. The percentage of DiD-positive acceptor cells in the total cell population was evaluated. Data represent the mean (±SEM), normalized to control cells arbitrarily set at 100%, of at least 4 independent experiments. *P < 0.05; **P < 0.01; ***P < 0.001; ns = not significant.

To address the functional relevance of CDC42, IRSp53, and VASP action on TNT formation, we investigated their effects on TNT-mediated vesicle transfer. We used a well-established protocol that allows the evaluation of cell-contact dependent intercellular vesicle transfer^24^,^30^,^43^. This protocol is based on the co-culture of two cell populations with a 1:1 ratio: a “donor population”, whose internal vesicles are labeled with the non-specific membrane dye DiD, and an “acceptor population”, transfected with a different fluorescent marker (H2B-mCherry or H2B-GFP). After 16 h of co-culture, the cells are fixed and the number of acceptor cells having received DiD-labeled vesicles from the donor population is evaluated by flow cytometry. The cell-contact requirement of this transfer was assessed by using a filter to physically separate the two cell populations while allowing the transfer of exosomes and soluble molecules or by applying the conditioned medium from the donor cells onto the acceptor population for the same duration as the co-culture, as we previously showed^30^. After transfecting the donor population with GFP-CDC42 V12, RFP-IRSp53, and GFP-VASP, we observed a significant reduction in intercellular vesicle transfer (40%, 15% and 20% on average, respectively) (Fig. 2a and b, Supplementary Fig. 3). On the other hand, we observed no significant difference in comparison to control group upon overexpression of GFP-CDC42 T17N or RFP-IRS FP/AA (Fig. 2a and b, Supplementary Fig. 3). These effects on vesicle transfer paralleled those seen on the number of TNT-connected cells indicating that CDC42, IRSp53, and VASP act as negative regulators of both TNT formation and transfer function.

### CDC42, IRSp53, and VASP act as a network to inhibit TNT formation

**Figure 3:**
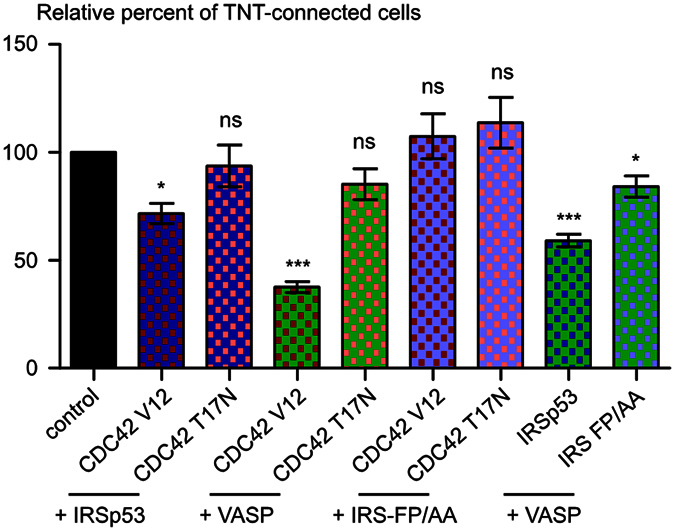
CDC42, IRSp53, and VASP act as a network to negatively regulate the number of TNT-connected cells. CAD cells were transiently co-transfected as indicated under the graph. Cells were fixed 14 hrs post-transfection, labeled with fluorescent WGA in order to detect TNTs and observed by confocal microscopy. The ratio of TNT-forming cotransfected cells/number of cotransfected cells was evaluated. Data represent the mean (±SEM), normalized to control cells arbitrarily set at 100%, of at least 4 independent experiments. *P < 0.05; **P < 0.01; ***P < 0.001; ns = not significant.

Next, we investigated whether the mechanism by which CDC42, IRSp53, and VASP inhibit TNT formation involved them acting independently or in concert. We co-transfected cells with either CDC42/IRSp53, IRSp53/VASP or CDC42/VASP couples of different constructs described above, and evaluated the number of TNT-forming cells by immunofluorescence. Interestingly, co-transfection of the dominant negative form of CDC42, (GFP-CDC42-T17N and Myc-CDC42 T17N), RFP-IRSp53, or GFP-VASP reverted the decrease in TNT-connected cells observed upon transfection of either RFP-IRSp53 or GFP-VASP alone. Notably, this finding indicates that CDC42 activity is required for IRSp53 and VASP inhibition of TNT formation (Fig. 3, Supplementary Fig. 4). On the other hand, the IRSp53 mutant (RFP-IRS FP/AA) reverted the decrease in TNT-connected cells induced by GFP-CDC42 V12 overexpression, suggesting that CDC42 negative regulation of TNT formation requires IRSp53 capacity to recruit its protein partners (Fig. 3, Supplementary Fig. 4). Upon co-expression of GFP-VASP and RFP-IRSp53, we observed a significantly higher reduction in the number of TNT-connected cells compared to the decrease observed after the expression of either GFP-VASP or RFP-IRSp53 alone (Fig. 3, Supplementary Fig. 4). Collectively, these results indicate that CDC42, IRSp53, and VASP act as a network which negatively regulates TNT formation.

### VASP positively regulates filopodia formation

**Figure 4:**
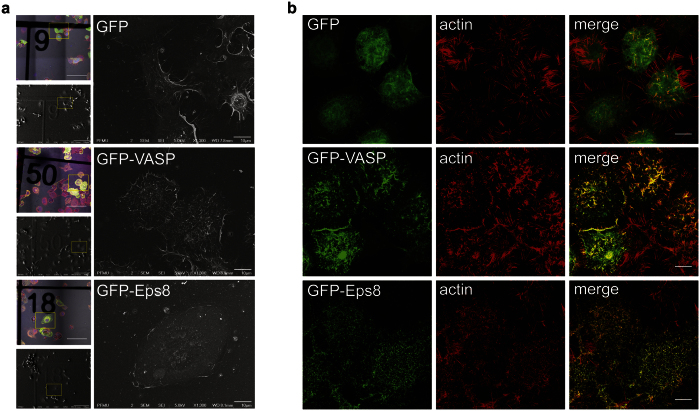
VASP and Eps8 ectopic expression have opposite effect on filopodia formation. (**a**) Morphology of CAD cells expressing GFP-VASP or GFP-Eps8 characterized by CL-SEM. Cells were transiently transfected with GFP-VASP, GFP-Eps8 or GFP as a control. Cells were plated for 6 hrs on correlative microscopy coverslips, fixed and stained with WGA Alexa Fluor^©^-594 nm. Coverslips were first imaged by confocal microscopy, then prepared for SEM and imaged with a JEOL JSM 6700 F field emission scanning electron microscope. Scale bar = 10 μM. (**b**) Morphology of CAD cells transfected with GFP-VASP or GFP-Eps8 characterized by SR-SIM. Cells were fixed after 14 hrs. Actin was stained using rhodamine-phalloidin. Cells were imaged by SR-SIM using an Elyra P.S.1 microscope. Scale bar = 10 μM.

Because the CDC42/IRSp53/VASP network was shown to act as a positive regulator in filopodia formation in neuronal cells^36^,^38^,^44^, we analyzed whether it plays a similar role in CAD cells. However, since CDC42 and IRSp53 can also reduce the formation of membrane ruffles and lamellipodia that can mask filopodia formation we focused on VASP. VASP was shown to act downstream of IRSp53 for filopodia induction, but is not involved in IRSp53-mediated lamellipodia formation^38^,^44^. The formation of filopodia was qualitatively assessed by both correlative-scanning electron microscopy (CL-SEM) (Fig. 4a) and super resolution structured illumination microscopy (SR-SIM) imaging (Fig. 4b) of transfected cells. In most cases, VASP transfected cells exhibited an increase in the number of actin-positive, filopodia-like dorsal protrusions with respect to GFP transfected control cells, suggesting that VASP also exerts a positive regulation of filopodia formation in CAD cells. Contrary to TNTs and dorsal filopodia, attached filopodia display vinculin-positive focal adhesion at their tips^33^,^45^. We used this specificity to automatically detect and count vinculin-positive peripheral cellular protrusions, in order to obtain more quantitative data on the role of VASP in attached filopodia formation (see methods)^46^. We demonstrated an enrichment of peripheral focal adhesions in GFP-VASP transfected cells compared to the control, confirming the involvement of VASP in promoting filopodia formation in our cell line model (Supplementary Fig. 5).

### Eps8 positively regulates TNT formation and function via its bundling activity

The results presented above show that positive regulators of filopodia formation can inhibit TNT formation and function. This suggests that TNTs and filopodia might be distinct structures oppositely regulated by the same signaling pathways. To further investigate this hypothesis, we decided to look at the role in TNT formation of Eps8, another actin regulator shown to negatively regulate the formation of filopodia in neuronal cells^40^. First, the endogenous levels of Eps8 were analyzed in CAD cells (Supplementary Fig. 6). We found that CAD cells express very low amounts of Eps8 mRNA, compared to brain tissue and CHO cells, and the protein was not detectable, thus the role of this actin regulator was studied by its overexpression. Consistent with data present in the literature in other cell models, we observed a nearly complete disappearance of dorsal and peripheral filopodia like protrusions in CAD cells overexpressing GFP-Eps8 (Fig. 4a and b). Indeed the CL-SEM (Fig. 4a) and SR-SIM (Fig. 4b) images showed tiny actin-positive protrusions, reminiscent to the club-like protrusions previously described in neurons overexpressing this construct^40^.

**Figure 5:**
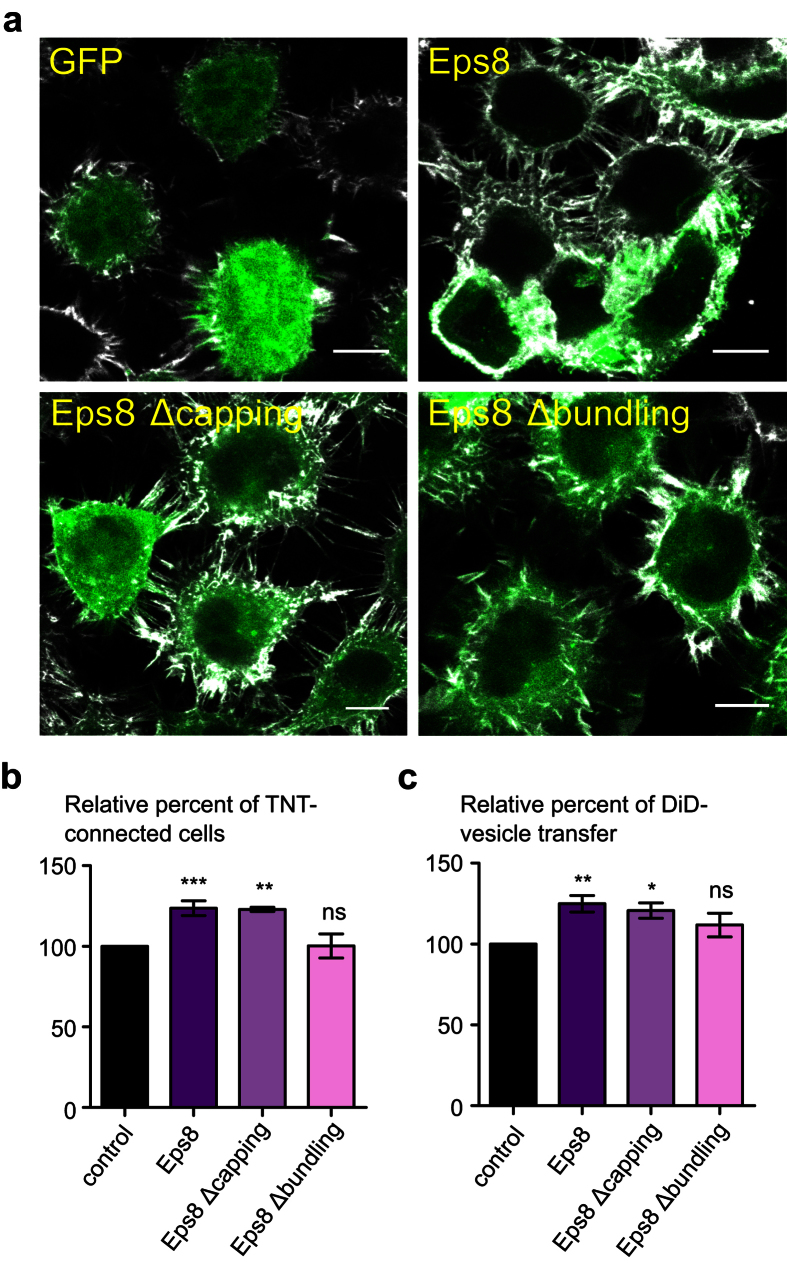
Eps8 positively regulates TNT formation and intercellular vesicle transfer via its bundling activity. (**a**) Representative confocal images showing intercellular connections upon ectopic expression of GFP-Eps8, GFP-Eps8Δcapping or GFP-Eps8Δbundling. Cells were fixed 14 hrs post-transfection, labeled with WGA-Alexa Fluor^©^-647 nm (grey) in order to detect TNTs and observed by confocal microscopy. Scale bar = 10 μM. (**b**) Quantification of TNT-connected cells upon ectopic expression of GFP-Eps8, GFP-Eps8Δcapping or GFP-Eps8Δbundling. The ratio of TNT-forming transfected cells/number of transfected cells was evaluated. (**c**) The donor population of CAD cells was transfected with either GFP-Eps8, GFP-Eps8Δcapping or GFP-Eps8Δbundling, and the acceptor population was transfected with H2B-mCherry. The co-culture was prepared as described in Fig. 3. Cells were analyzed by flow cytometry to quantify the percentage of acceptor cells having received DiD-labeled vesicles from the donor population. Data represent the mean (±SEM), normalized to control cells arbitrarily set at 100%, of at least 3 independent experiments. (**b**,**c**) Data represent the mean (±SEM), normalized to control cells (GFP-vector transfected cells) arbitrarily set at 100%, of at least 3 independent experiments. *P < 0.05; **P < 0.01; ***P < 0.001; ns = not significant.

Intriguingly, ectopic expression of GFP-Eps8 in CAD cells induced a significant increase in the number of TNT-connected cells (Fig. 5a and b, Supplementary Fig. 7). To dissect the mechanism of Eps8 action, we assessed the effect on TNT formation of its actin-capping and -bundling activity. We transfected CAD cells with GFP-Eps8 Δcapping and GFP-Eps8 Δbundling, (constructs encoding Eps8 mutants impaired in their actin-capping and -bundling activity respectively)^47^. The expression of GFP-Eps8 Δcapping-mutant, likewise of Eps8, increased TNT-connected cells. Conversely, no increase upon the expression of GFP-Eps8 Δbundling-mutant was observed (Fig. 5a and b, Supplementary Fig. 7). This finding indicates that Eps8 exerts a positive regulation on TNT formation via its bundling activity. We, then, evaluated whether Eps8 had any functional consequences by looking at the effect on intercellular vesicle transfer. We observed a significant increase of DiD-positive acceptor cells upon expression of GFP-Eps8 or GFP-Eps8 Δcapping compared to the control, whereas no significant change was monitored for GFP-Eps8 Δbundling (Fig. 5c, Supplementary Fig. 7). Overall this data indicates that Eps8 is a positive regulator of functionally active TNTs and that its actin-bundling activity is required.

## Discussion

**Figure 6:**
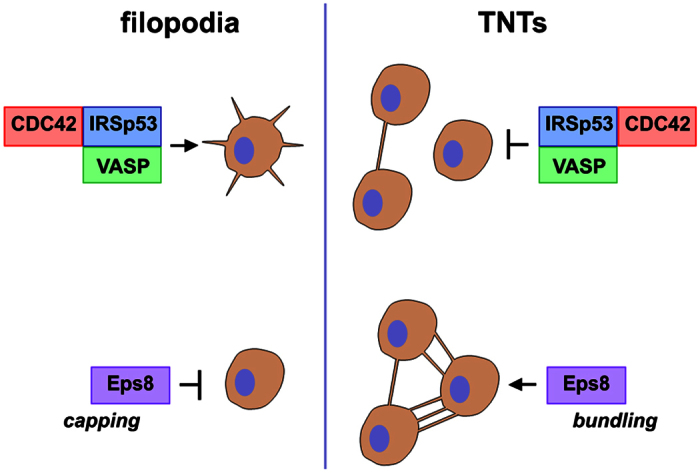
TNT and filopodia formation involve the same actin regulatory complexes acting in opposite ways.

The data presented here provides new insights on the cytoskeleton architecture and mechanisms underlying TNT formation and intercellular vesicle transfer in neuronal CAD cells. Our results show that CDC42, IRSp53, and VASP act in concert to inhibit TNT formation while concomitantly promoting the extension of filopodia (Fig. 6). We previously observed a drastic reduction of TNT-connected cells upon overexpression of GFP-VASP in CAD cells^30^. Here, we demonstrate that VASP expression also reduces contact-dependent intercellular vesicle transfer, supporting a negative role of VASP on functional TNTs. Our data also points to IRSp53 as another negative regulator of TNT formation and transfer. To exert this function, IRSp53 must interact with downstream partners since no decrease in TNT-connected cells nor vesicle transfer were observed upon overexpression of a mutant form of IRSp53 impaired in binding its downstream partners (eg, VASP, N-WASP, Wave, mDia and Eps8^32^). Nevertheless, we observed a stronger inhibition of TNT formation upon co-expression of IRSp53 and VASP compared to the expression of one or the other alone. This indicates that VASP might cooperate with IRSp53 (perhaps by acting downstream as proposed in filopodia^36^), although we cannot exclude that IRSp53 inhibition of TNT formation and function also involves additional interacting partners. More importantly, we showed that activation of CDC42 negatively regulates TNT formation and transfer function. Indeed, while the constitutively active form of the GTPase strongly reduces the number of cells connected via TNTs and intercellular vesicle transfer, overexpression of the dominant-negative form increases TNT formation without a significant effect on vesicle transfer. This latter observation suggests that the formation of functional TNTs requires additional limiting factors. It is important to note that both IRSp53- and VASP-mediated inhibition of TNT formation and transfer appears to require CDC42 activity, suggesting that an active CDC42-IRSp53-VASP complex is needed to counteract TNT formation and activity.

Our data contradicts prior work showing a decrease in TNT formation and elongation upon inhibition of CDC42^26^,^27^,^41^. The contact-dependent intercellular transfer was also shown to require CDC42 activity^48^,^49^. However, all of these studies were performed in non-neuronal cells (Hela, Jurkat and NK cells). Thus, this apparent discrepancy could simply reflect cell type-dependent mechanisms of TNT formation, as it has been reported for M-Sec and p53-dependent mechanisms^29^,^30^. It is also worth pointing out that IRSp53, VASP, and Eps8 display context-dependent effects on filopodia formation that may be directly relevant in the context of TNT formation and could account, in part, to an specific cell activity of this actin regulatory signaling axis (see below)^36^,^38^.

The CDC42/IRSp53/VASP network has previously been identified as a positive regulator of filopodia formation^36^,^38^. In CAD cells, we observed an increase of actin-rich dorsal protrusions and peripheral vinculin-positive focal adhesions upon overexpression of GFP-VASP. This indicates that VASP also positively regulates filopodia formation in our cellular model. On the other hand, the inhibitory role of the CDC42/IRSp53/VASP network in TNT formation reported here suggests that filopodia and TNTs are distinct structures that utilize different mechanisms for their formation (Fig. 6). In order to investigate this hypothesis, we study the role of Eps8. Eps8 can coordinate and integrate multiple signaling pathways via the formation of distinct macromolecular complexes^50^,^51^. It is involved in the endocytosis of receptor tyrosine kinases and can transduce signals from Ras to Rac, leading to actin remodeling^51^. Eps8 directly interacts with actin and is capable of capping and bundling actin filaments depending on its downstream ligands^47^. In keeping with the latter notion, Eps8 was shown to play a complex role in filopodia regulation^38^. It acts as a positive regulator of filopodia formation in non-neuronal cells (Hela and epithelial cells), where it was shown to synergize with IRSp53 in bundling actin filaments^38^,^52^. On the other hand, the genetic removal of Eps8 increases the formation of filopodia in primary hippocampal neurons^38^,^40^. This inhibitory function is exerted, instead, through the capping activity of Eps8 triggered by the interaction with Abi1/2, and by its competition with VASP-family proteins for IRSp53 binding. In CAD cells, we observed a striking remodeling of cellular protrusions upon overexpression of GFP-Eps8. Instead of the abundant peripheral and dorsal filopodia-like protrusions observed in control cells, GFP-Eps8-transfected cells harbor tiny and curved actin-rich protrusions. This phenotype may correspond to the thick club-like protrusions observed along axon and dendrites in primary neurons upon ectopic expression of Eps8^40^. These observations suggest that Eps8 negatively regulates filopodia formation in our cellular model as well as in primary neurons. Interestingly, Eps8 overexpression induced a significant increase in TNT-connected cells and intercellular vesicle transfer, indicating that it may act as a positive regulator of TNT formation. Our data also indicates that Eps8-mediated TNT induction involves the bundling activity of the protein, while the capping activity does not seem to be required. However, it was previously shown that capping activity of Eps8 is regulated by MAPK-dependent phosphorylation, suggesting that modulation of this latter activity rather than an on/off switch may be important in controlling actin-based structures in filopodia as well as in TNTs^53^. Most of the data published in the literature so far points to a synergy with IRSp53, driving Eps8 bundling activity^38^,^52^. However, IRSp53 is unlikely to contribute to Eps8-dependent TNT induction in CAD cells as it is a negative regulator of TNT formation. Further work will have to focus on the identification of Eps8 partners in this context.

In summary, our results represent the first molecular evidence that TNTs and filopodia may be completely different structures, which invalidates the hypothesis that TNTs could arise from a subset of filopodia. In fact, our data rather suggests that TNT precursors are different structures from the beginning. Furthermore, the observation that the same actin regulators may be involved in filopodia and TNT formation, but acting in opposite ways, raises the intriguing possibility of a “switch” between the two structures. Interestingly, our laboratory previously reported that when a growing TNT-like protrusion attaches to a distant cell, the filopodia of this cell subsequently retracts^30^. This constitutes an additional hint for a regulated balance between TNTs and filopodia. Whether this balance results from a signaling pathway having a physiological relevance, or if it is the consequence of a limiting factor, remains to be determined but nevertheless calls for further studies both at the molecular and ultrastructural level.

## Methods

### Cell culture, transfection and DNA constructs

Mouse neuronal CAD cells were kindly given by Hubert Laude (Institut National de la Recherche Agronomique, Jouy-en-Josas, France) and were cultured in Gibco^TM^ Opti-MEM^©^ (Invitrogen) supplemented with 10% fetal bovine serum and 1% penicillin/streptomycin. Transient transfections were performed with Lipofectamine 2000 (Invitrogen) in accordance with the manufacturer’s instructions. The various GFP-tagged Eps8 constructs were previously described^47^,^52^. RFP-IRSp53 and RFP-IRS FP/AA were a kind gift from Sohail Ahmed (IMB, Singapore). GFP-CDC42 V12 and GFP-CDC42 T17N were a gift from Roberto Mayor (University College London, UK) and GFP-VASP was a gift from Sandrine Etienne-Manneville (Pasteur Institute, Paris, France). Myc-CDC42 V12 and Myc-CDC42 T17N were obtained from Nathalie Sauvonnet (Pasteur Institute, Paris, France). H2B-GFP was a gift from Geoff Wahl (Addgene plasmid # 11680^54^), and H2B-mCherry was a gift from Robert Benezra (Addgene plasmid # 20972^55^). Myc-tagged mouse Rac1 17 N and Rac1 V12 were a kind gift from Dr. A Hall from the University College London, UK. Visualization of Rac1-Myc-tagged cells was performed using a c-Myc mouse monoclonal antibody (Santa Cruz) in a 1:100 dilution, followed by a goat anti-mouse secondary antibody Alexa Fluor©-546 in a 1:500 dilution, both dissolved in a 2% BSA in PBS solution.

### Quantification of TNT-connected cells

Confluent CAD cells were mechanically detached and counted, and 100,000 cells were plated for 24 hrs on Ibidi μ-dishes (Biovalley, France). Cells were transfected as described above, with the appropriate plasmids. At 14 hrs post-transfection, cells were fixed for 15 min at 37 °C in 2% PFA, 0.05% glutaraldehyde and 0.2 M HEPES in PBS, and then additionally fixed for 15 min in 4% PFA and 0.2 M HEPES in PBS. Cells were carefully washed in PBS, labeled for 20 min at RT with a 3.3 μg.μL^−1^ solution of Wheat Germ Agglutinin (WGA) Alexa Fluor^©^-647 nm conjugate (Invitrogen) in PBS, washed again and sealed with Aqua-Poly/Mount (Polysciences, Inc.). The whole cellular volume was imaged by acquiring 0.5 μm Z-stacks with an inverted confocal microscope (Zeiss LSM 700) controlled by ZEN software. TNT-connected cells, *i.e.* cells connected by straight WGA-labeled structures that do not touch the substrate and have a diameter smaller than 1 μm were manually counted by experimenters blind to the condition in the same manner as previously described^20^,^23^,^30^. Displayed images correspond to stack projections. Only linear corrections were applied using the software ImageJ.

### Quantification of vinculin-positive peripheral focal adhesion

For indirect immunofluorescence labeling of vinculin, 90,000 cells were plated for 16 hrs on Ibidi μ-dishes and then fixed in 4% PFA in PBS for 15 min at 37 °C. Samples were quenched with 50 mM NH_4_Cl for 15 min, then cells were permeabilized with 0.01% saponin in PBS containing 2% BSA (w/v) for 20 min at 37 °C. After a first 1 hr incubation with mouse anti-vinculin antibody (V9264, Sigma) diluted 1:500 in PBS containing 0.01% saponin and 2% BSA (w/v), cells were thoroughly washed and incubated for 40 min with goat anti-mouse AlexaFluor^©^-488 (Invitrogen) diluted 1:500 in PBS containing 0.01% saponin and 2% BSA (w/v). Cells were washed and sequentially stained for 20 min with a 3.3 μg.μL^−1^ solution of WGA Alexa Fluor^©^-647 nm conjugate, for 30 min with a 1 μg.mL^−1^ solution of HCS CellMask^TM^ Blue, which stains the entire cell volume (i.e. cytoplasm and nucleus), and for 5 min with a 0.2 μg.μL^−1^ solution of DAPI. Samples were washed and sealed with Aqua-Poly/Mount (Polysciences, Inc.). The bottom of the cell (in contact with the plastic dish) was imaged with an inverted confocal microscope (Zeiss LSM700) controlled by ZEN software. Displayed images correspond to stack projections. Only linear corrections were applied, using the software ImageJ. Vinculin-positive peripheral focal adhesion were automatically detected and counted using ICY software (http://icy.bioimageanalysis.org/).

### Quantification of vesicle intercellular transfer

CAD cells were separately transfected in T25 flasks with the appropriate constructs (donor cells) and H2B-mCherry or H2B-GFP (acceptor cells) for 3 hrs in serum-free medium and incubated for one additional hour in complete medium. Donor cells were detached, counted and labeled with a 333 nM solution of the lypophilic tracer Vybrant^TM^ DiD (long-chain dialkylcarbocyanine) in complete medium for 30 min at 37 °C. Cells were pelleted at 1000 rpm for 4 min to remove the DiD solution, resuspended in complete medium and incubated for 45 min at 37 °C in order to internalize the dye. Cells were then pelleted once more to wash away any remaining dye and resuspended in complete medium. The labeled donor cells were mixed in a 1:1 ratio with H2B-transfected acceptor cells and plated at subconfluence (120,000 cells per well) on 24-well plates for 16 hrs at 37 °C. Each independent co-culture was performed in triplicate. Cells were then washed with PBS to remove any dead cells, mechanically detached from the dish by pipetting up and down with 500 μl PBS, and passed through sterile 40-mm nylon cell strainers (BD Falcon^TM^) in order to obtain single-cell suspensions. Cell suspensions were fixed with 500 μl of 4% PFA (2% final solution). Flow cytometry data were acquired using a LSR Fortessa flow cytometer (BD Biosciences). GFP fluorescence was analyzed at 488 nm excitation wavelength, RFP and mCherry fluorescence were analyzed at 561 nm excitation wavelength, and DiD fluorescence was analyzed at 640 nm excitation wavelength. Samples were analyzed at high flow rate, corresponding to 200–400 events per second and 10,000 events were acquired for each condition. The data were analyzed using FlowJo analysis software.

### Correlative scanning electron microscopy

CADs were transfected as described above with GFP, GFP-VASP or GFP-Eps8 and 500,000 cells were plated on sterile (UV sterilization) plastic Correlative Microscopy Coverslips^©^ (10 × 10 grids of 1 mm squares, 66108-03, EMS). After 6 hrs at 37 °C, cells were fixed for 20 min at 37 °C in 2% PFA, 0.05% GA and 0.2 M HEPES in PBS, then additionally fixed for 20 min in 4% PFA and 0.2 M HEPES in PBS. Cells were carefully washed in PBS, labeled for 20 min at RT with a 3.3 μg.μL^−1^ solution of WGA Alexa Fluor^©^-594 nm or -488 nm conjugate (Invitrogen) in PBS, then washed again. Samples were imaged in PBS using an upright LSM700 confocal microscope (Zeiss) with an immersed long-distance working 40X objective. Cells were then fixed a second time in 2.5% GA in PHEM (pH 6.9). They were washed three times with 0.2 M cacodylate buffer (pH 7.2), post-fixed for 1 hr in 1% (w/v) osmium tetroxide in 0.1 M cacodylate buffer (pH 7.2), and then rinsed with distilled water. Samples were dehydrated through a graded series of 25, 50, 17 and 95% ethanol solution for 5 min each time. Samples were then dehydrated for 10 min in 100% ethanol, followed by critical point drying with CO_2_. Dried specimens were spattered with 10 nm gold palladium, with a GATAN Ion Beam Coater and were examined and photographed with a JEOL JSM 6700 F field emission scanning electron microscope operating at 5Kv. Images were acquired with the upper SE detector (SEI). Only linear corrections were applied, using Image J software.

### Super Resolution Structured Illumination Microscopy (SR-SIM)

Cells were prepared as described for the quantification of TNT-connected cells. Samples were imaged with an Elyra P.S.1 microscope controlled by ZEN software, using a 63 × 1.4NA plan-apo objective lens. Data sets were collected with five grating phases, three rotations and 300 nm Z-stacks. SIM post-processing was performed using the ZEN software in manual mode to optimize noise filtering and sectioning parameters for each channel. Reconstructed images were then corrected for spatial misalignment between spectrally distinct channels (variations in the dichroic mirrors) with the Channel Alignment method of the ZEN software. Polystyrene beads coated with multiple fluorophores were used for calibration. Displayed images correspond to stack projections. Only linear corrections were applied, using Image J software.

### Statistical analysis

Mean comparisons were calculated on the raw data by two-tailed paired Student’s t-test using the software Prism. Normal distributions were assumed but not formally tested. All experiments were repeated at least three times and no issues in reproducibility were encountered.

### Western blot

Mouse brain tissue and cells were lysed in NP-40 lysis buffer (25 mM Tris pH 7.5, 150 mM NaCl, 1% NP-40), and protein concentration in the cell lysate was quantified using a Bradford protein assay (Bio-Rad). Protein samples were incubated at 100 °C for 5 min and electrophoresed on 10% SDS-polyacrylamide gels. Proteins were transferred onto PVDF membranes (GE Healthcare Life sciences). Membranes were blocked in 5% nonfat milk in Tris-buffered saline with 0.1% Tween 20 (TBS-T) for 1 h. Membranes were then incubated at 4 °C with a primary antibody mouse anti-Eps8 (BD Transduction) and mouse anti-GAPDH (Millipore) diluted in 5% nonfat milk overnight (1:500 and 1:10,000, respectively) then washed several times with TBS-T. After 1 h incubation with horseradish peroxidase-conjugated anti-mouse IgG secondary antibody (1:10,000) (GE Healthcare Life sciences), membranes were washed with TBS-T and protein bands on the membrane were detected using an ECL-Plus immunoblotting chemiluminescence system (GE Healthcare Life sciences). Membranes were imaged using ImageQuant LAS 500TM camera (GE Healthcare Life sciences).

### RNA preparation and Reverse Transcription PCR

Total RNA was prepared from CAD and CHO cells in 25-mm dishes with an RNeasy Plus Micro Kit (QIAGEN Cat: 74034) according to the manufacturer’s instructions. Reverse transcription-PCR was performed using random hexamer primers, and using a SuperScript II Reverse Transcriptase kit (Invitrogen). The following primers were used for Eps8: a forward primer sequence: 5′- caatgtgtccgactatcctc -3′, and a reverse primer sequence: 5′- tcagtggctgctcccttcat -3′. A total of 2 μL of the product was used to amplify a fragment of Eps8 cDNA for 30 cycles.

## Additional Information

**How to cite this article**: Delage, E. *et al*. Differential identity of Filopodia and Tunneling Nanotubes revealed by the opposite functions of actin regulatory complexes. *Sci. Rep.* **6**, 39632; doi: 10.1038/srep39632 (2016).

**Publisher's note:** Springer Nature remains neutral with regard to jurisdictional claims in published maps and institutional affiliations.

## Supplementary Material

Supplementary Information

## References

[b1] Rustom A., Saffrich R., Markovic I., Walther P. & Gerdes H. H. Nanotubular highways for intercellular organelle transport. Science 303, 1007–1010, doi: 10.1126/science.1093133 (2004). 14963329

[b2] Abounit S. & Zurzolo C. Wiring through tunneling nanotubes–from electrical signals to organelle transfer. Journal of cell science 125, 1089–1098, doi: 10.1242/jcs.083279 (2012). 22399801

[b3] Marzo L., Gousset K. & Zurzolo C. Multifaceted roles of tunneling nanotubes in intercellular communication. Frontiers in physiology 3, 72, doi: 10.3389/fphys.2012.00072 (2012). 22514537 PMC3322526

[b4] Chinnery H. R., Pearlman E. & McMenamin P. G. Cutting edge: Membrane nanotubes *in vivo*: a feature of MHC class II+ cells in the mouse cornea. Journal of immunology 180, 5779–5783 (2008). 10.4049/jimmunol.180.9.5779 PMC3392179 18424694

[b5] Lou E. . Tunneling nanotubes provide a unique conduit for intercellular transfer of cellular contents in human malignant pleural mesothelioma. PloS one 7, e33093, doi: 10.1371/journal.pone.0033093 (2012). 22427958 PMC3302868

[b6] Pasquier J. . Preferential transfer of mitochondria from endothelial to cancer cells through tunneling nanotubes modulates chemoresistance. Journal of translational medicine 11, 94, doi: 10.1186/1479-5876-11-94 (2013). 23574623 PMC3668949

[b7] Seyed-Razavi Y., Hickey M. J., Kuffova L., McMenamin P. G. & Chinnery H. R. Membrane nanotubes in myeloid cells in the adult mouse cornea represent a novel mode of immune cell interaction. Immunology and cell biology 91, 89–95, doi: 10.1038/icb.2012.52 (2013). 23146944

[b8] Gerdes H. H., Rustom A. & Wang X. Tunneling nanotubes, an emerging intercellular communication route in development. Mechanisms of development 130, 381–387, doi: 10.1016/j.mod.2012.11.006 (2013). 23246917

[b9] Hashimoto M. . Potential Role of the Formation of Tunneling Nanotubes in HIV-1 Spread in Macrophages. Journal of immunology 196, 1832–1841, doi: 10.4049/jimmunol.1500845 (2016). 26773158

[b10] Sowinski S. . Membrane nanotubes physically connect T cells over long distances presenting a novel route for HIV-1 transmission. Nature cell biology 10, 211–219, doi: 10.1038/ncb1682 (2008). 18193035

[b11] Onfelt B. . Structurally distinct membrane nanotubes between human macrophages support long-distance vesicular traffic or surfing of bacteria. Journal of immunology 177, 8476–8483 (2006). 10.4049/jimmunol.177.12.8476 17142745

[b12] Lou E. Intercellular conduits in tumours: the new social network. Trends in cancer 2, 3–5, doi: 10.1016/j.trecan.2015.12.004 (2016). PMC4776333 26949744

[b13] Ware M. J. . Radiofrequency treatment alters cancer cell phenotype. Scientific reports 5, 12083, doi: 10.1038/srep12083 (2015). 26165830 PMC4499808

[b14] Gousset K. & Zurzolo C. Tunnelling nanotubes: a highway for prion spreading? Prion 3, 94–98 (2009). 19471116 10.4161/pri.3.2.8917 PMC2712606

[b15] Langevin C., Gousset K., Costanzo M., Richard-Le Goff O. & Zurzolo C. Characterization of the role of dendritic cells in prion transfer to primary neurons. The Biochemical journal 431, 189–198, doi: 10.1042/BJ20100698 (2010). 20670217

[b16] Victoria G. S., Arkhipenko A., Zhu S., Syan S. & Zurzolo C. Astrocyte-to-neuron intercellular prion transfer is mediated by cell-cell contact. Scientific reports 6, 20762, doi: 10.1038/srep20762 (2016). 26857744 PMC4746738

[b17] Zhu S., Victoria G. S., Marzo L., Ghosh R. & Zurzolo C. Prion aggregates transfer through tunneling nanotubes in endocytic vesicles. Prion 9, 125–135, doi: 10.1080/19336896.2015.1025189 (2015). 25996400 PMC4601206

[b18] Costanzo M. . Transfer of polyglutamine aggregates in neuronal cells occurs in tunneling nanotubes. Journal of cell science 126, 3678–3685, doi: 10.1242/jcs.126086 (2013). 23781027

[b19] Wang Y., Cui J., Sun X. & Zhang Y. Tunneling-nanotube development in astrocytes depends on p53 activation. Cell death and differentiation 18, 732–742, doi: 10.1038/cdd.2010.147 (2011). 21113142 PMC3131904

[b20] Abounit S. . Tunneling nanotubes spread fibrillar alpha-synuclein by intercellular trafficking of lysosomes. The EMBO journal 35, 2120–2138, doi: 10.15252/embj.201593411 (2016). 27550960 PMC5048354

[b21] Abounit S., Wu J. W., Victoria G. S. & Zurzolo C. Tunneling nanotubes: A possible highway in the spreading of tau and other prion-like proteins in neurodegenerative diseases. Prion 0, doi: 10.1080/19336896.2016.1223003 (2016). PMC5105909 27715442

[b22] Costanzo M. & Zurzolo C. The cell biology of prion-like spread of protein aggregates: mechanisms and implication in neurodegeneration. The Biochemical journal 452, 1–17, doi: 10.1042/BJ20121898 (2013). 23614720

[b23] Abounit S., Delage E. & Zurzolo C. Identification and Characterization of Tunneling Nanotubes for Intercellular Trafficking. Current protocols in cell biology/editorial board, Juan S. Bonifacino … [et al.] 67, 12 10 11-21, doi: 10.1002/0471143030.cb1210s67 (2015). 26061240

[b24] Bukoreshtliev N. V. . Selective block of tunneling nanotube (TNT) formation inhibits intercellular organelle transfer between PC12 cells. FEBS letters 583, 1481–1488, doi: 10.1016/j.febslet.2009.03.065 (2009). 19345217

[b25] Gousset K. . Prions hijack tunnelling nanotubes for intercellular spread. Nature cell biology 11, 328–336, doi: 10.1038/ncb1841 (2009). 19198598

[b26] Hase K. . M-Sec promotes membrane nanotube formation by interacting with Ral and the exocyst complex. Nature cell biology 11, 1427–1432, doi: 10.1038/ncb1990 (2009). 19935652

[b27] Schiller C. . LST1 promotes the assembly of a molecular machinery responsible for tunneling nanotube formation. Journal of cell science 126, 767–777, doi: 10.1242/jcs.114033 (2013). 23239025

[b28] Takahashi A. . Tunneling nanotube formation is essential for the regulation of osteoclastogenesis. Journal of cellular biochemistry 114, 1238–1247, doi: 10.1002/jcb.24433 (2013). 23129562

[b29] Andresen V. . Tunneling nanotube (TNT) formation is independent of p53 expression. Cell death and differentiation 20, 1124, doi: 10.1038/cdd.2013.61 (2013). 23764777 PMC3705610

[b30] Gousset K., Marzo L., Commere P. H. & Zurzolo C. Myo10 is a key regulator of TNT formation in neuronal cells. Journal of cell science 126, 4424–4435, doi: 10.1242/jcs.129239 (2013). 23886947

[b31] Lokar M., Iglic A. & Veranic P. Protruding membrane nanotubes: attachment of tubular protrusions to adjacent cells by several anchoring junctions. Protoplasma 246, 81–87, doi: 10.1007/s00709-010-0143-7 (2010). 20526853

[b32] Arjonen A., Kaukonen R. & Ivaska J. Filopodia and adhesion in cancer cell motility. Cell adhesion & migration 5, 421–430, doi: 10.4161/cam.5.5.17723 (2011). 21975551 PMC3218609

[b33] Bohil A. B., Robertson B. W. & Cheney R. E. Myosin-X is a molecular motor that functions in filopodia formation. Proceedings of the National Academy of Sciences of the United States of America 103, 12411–12416, doi: 10.1073/pnas.0602443103 (2006). 16894163 PMC1567893

[b34] Watanabe T. M., Tokuo H., Gonda K., Higuchi H. & Ikebe M. Myosin-X induces filopodia by multiple elongation mechanism. The Journal of biological chemistry 285, 19605–19614, doi: 10.1074/jbc.M109.093864 (2010). 20392702 PMC2885239

[b35] Zhang H. . Myosin-X provides a motor-based link between integrins and the cytoskeleton. Nature cell biology 6, 523–531, doi: 10.1038/ncb1136 (2004). 15156152

[b36] Disanza A. . CDC42 switches IRSp53 from inhibition of actin growth to elongation by clustering of VASP. The EMBO journal 32, 2735–2750, doi: 10.1038/emboj.2013.208 (2013). 24076653 PMC3801440

[b37] Chou A. M., Sem K. P., Wright G. D., Sudhaharan T. & Ahmed S. Dynamin1 is a novel target for IRSp53 protein and works with mammalian enabled (Mena) protein and Eps8 to regulate filopodial dynamics. The Journal of biological chemistry 289, 24383–24396, doi: 10.1074/jbc.M114.553883 (2014). 25031323 PMC4148866

[b38] Vaggi F. . The Eps8/IRSp53/VASP network differentially controls actin capping and bundling in filopodia formation. PLoS computational biology 7, e1002088, doi: 10.1371/journal.pcbi.1002088 (2011). 21814501 PMC3140970

[b39] Kast D. J. . Mechanism of IRSp53 inhibition and combinatorial activation by Cdc42 and downstream effectors. Nature structural & molecular biology 21, 413–422, doi: 10.1038/nsmb.2781 (2014). PMC4091835 24584464

[b40] Menna E. . Eps8 regulates axonal filopodia in hippocampal neurons in response to brain-derived neurotrophic factor (BDNF). PLoS biology 7, e1000138, doi: 10.1371/journal.pbio.1000138 (2009). 19564905 PMC2696597

[b41] Arkwright P. D. . Fas stimulation of T lymphocytes promotes rapid intercellular exchange of death signals via membrane nanotubes. Cell research 20, 72–88, doi: 10.1038/cr.2009.112 (2010). 19770844 PMC2822704

[b42] Van Aelst L. & D’Souza-Schorey C. Rho GTPases and signaling networks. Genes & development 11, 2295–2322 (1997). 9308960 10.1101/gad.11.18.2295

[b43] Gurke S. . Tunneling nanotube (TNT)-like structures facilitate a constitutive, actomyosin-dependent exchange of endocytic organelles between normal rat kidney cells. Experimental cell research 314, 3669–3683, doi: 10.1016/j.yexcr.2008.08.022 (2008). 18845141

[b44] Lim K. B. . The Cdc42 effector IRSp53 generates filopodia by coupling membrane protrusion with actin dynamics. The Journal of biological chemistry 283, 20454–20472, doi: 10.1074/jbc.M710185200 (2008). 18448434

[b45] Schafer C. . The key feature for early migratory processes: Dependence of adhesion, actin bundles, force generation and transmission on filopodia. Cell adhesion & migration 4, 215–225 (2010). 20179423 10.4161/cam.4.2.10745 PMC2900617

[b46] Barzik M., McClain L. M., Gupton S. L. & Gertler F. B. Ena/VASP regulates mDia2-initiated filopodial length, dynamics, and function. Molecular biology of the cell 25, 2604–2619, doi: 10.1091/mbc.E14-02-0712 (2014). 24989797 PMC4148250

[b47] Hertzog M. . Molecular basis for the dual function of Eps8 on actin dynamics: bundling and capping. PLoS biology 8, e1000387, doi: 10.1371/journal.pbio.1000387 (2010). 20532239 PMC2879411

[b48] Biran A. . Senescent cells communicate via intercellular protein transfer. Genes & development 29, 791–802, doi: 10.1101/gad.259341.115 (2015). 25854920 PMC4403256

[b49] Frei D. M. . Novel microscopy-based screening method reveals regulators of contact-dependent intercellular transfer. Scientific reports 5, 12879, doi: 10.1038/srep12879 (2015). 26271723 PMC4536488

[b50] Cunningham D. L. . Novel binding partners and differentially regulated phosphorylation sites clarify Eps8 as a multi-functional adaptor. PloS one 8, e61513, doi: 10.1371/journal.pone.0061513 (2013). 23626693 PMC3634024

[b51] Di Fiore P. P. & Scita G. Eps8 in the midst of GTPases. The international journal of biochemistry & cell biology 34, 1178–1183 (2002). 12127568 10.1016/s1357-2725(02)00064-x

[b52] Disanza A. . Regulation of cell shape by Cdc42 is mediated by the synergic actin-bundling activity of the Eps8-IRSp53 complex. Nature cell biology 8, 1337–1347, doi: 10.1038/ncb1502 (2006). 17115031

[b53] Logue J. S. . Erk regulation of actin capping and bundling by Eps8 promotes cortex tension and leader bleb-based migration. eLife 4, e08314, doi: 10.7554/eLife.08314 (2015). 26163656 PMC4522647

[b54] Kanda T., Sullivan K. F. & Wahl G. M. Histone-GFP fusion protein enables sensitive analysis of chromosome dynamics in living mammalian cells. Current biology: CB 8, 377–385 (1998). 9545195 10.1016/s0960-9822(98)70156-3

[b55] Nam H. S. & Benezra R. High levels of Id1 expression define B1 type adult neural stem cells. Cell stem cell 5, 515–526, doi: 10.1016/j.stem.2009.08.017 (2009). 19896442 PMC2775820

