# Supplementary material for: Differential identity of Filopodia and Tunneling Nanotubes revealed by the opposite functions of actin regulatory complexes
Source: Sci Rep. 2016 Dec 23;6:39632. doi: 10.1038/srep39632 (PMC5180355; doi:10.1038/srep39632)
Supplement: Supplementary Information [file srep39632-s1.pdf]

Differential identity of Filopodia and Tunneling Nanotubes revealed by the opposite functions of actin regulatory complexes

AUTHORS :

Elise Delage[1], Diégo Cordero Cervantes[1,#], Esthel Pénard[1,#], Christine Schmitt[2], Sylvie Syan[1], Andrea Disanza[3], Giorgio Scita[3,4], Chiara Zurzolo[1*]

[1] Unité Trafic Membranaire et Pathogenèse, Institut Pasteur, 25-28 Rue du Docteur Roux, 75724 Paris CEDEX 15, France

[2] Ultrapole, Institut Pasteur, 25-28 Rue du Docteur Roux, 75724 Paris CEDEX 15, France

[3] FIRC Institute of Molecular Oncology, 20139 Milan, Italy

[4] Dipartimento di Scienze della Salute, Università degli Studi di Milano, 20122 Milan, Italy

[#] These authors contributed equally to this work

*corresponding author; e-mail: chiara.zurzolo@pasteur.fr

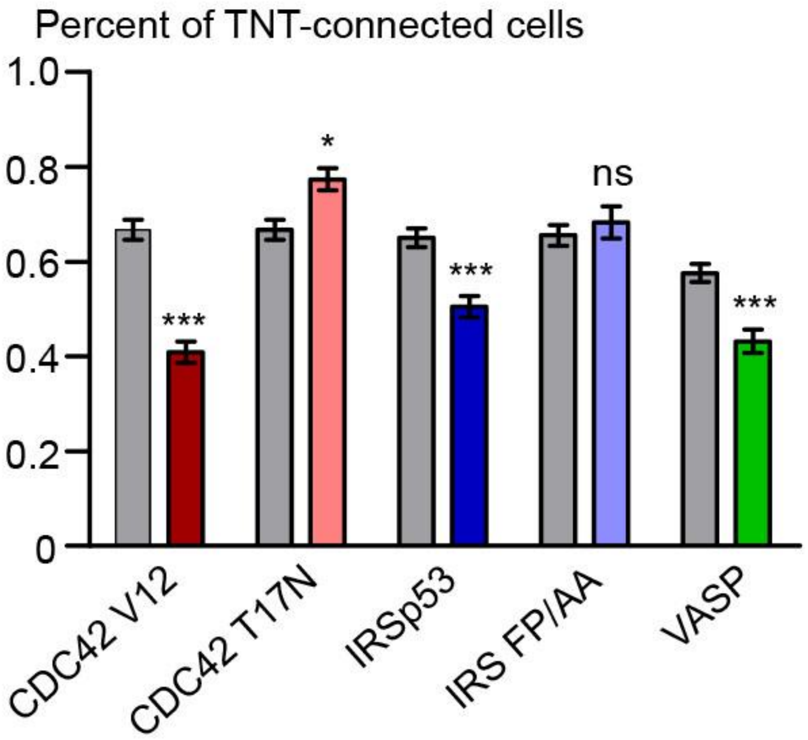

Supplementary Figure 1. CDC42, IRSp53, and VASP negatively regulate the number of TNT-connected cells.

Quantification of TNT-connected cells upon ectopic expression of GFP-CDC42 V12, GFP-CDC42 T17N, RFP-IRSp53, RFP-IRS FP/AA or GFPVASP (colored bars) compared to respective control (i.e. GFP or RFP, grey bars). The ratio of TNT-forming transfected cells/number of transfected cells was evaluated. Data represent the mean (±SEM) of at least 6 independent experiments. *P < 0.05; **P < 0.01; ***P < 0.001; ns = not significant.

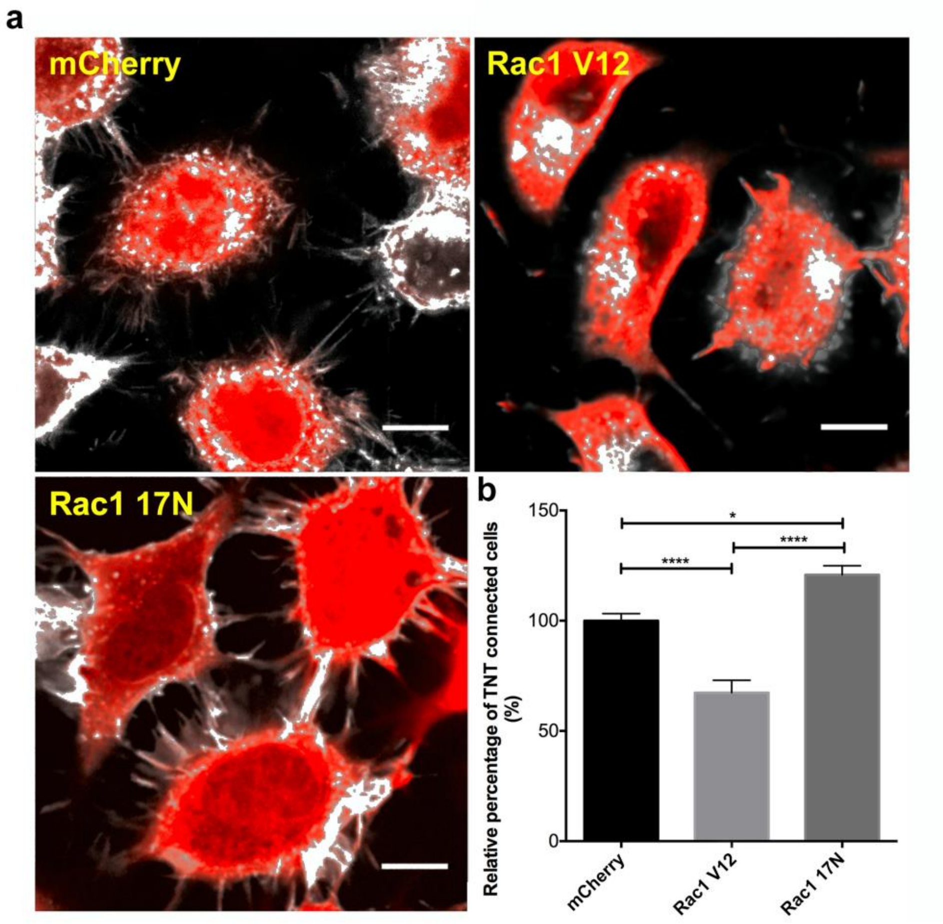

Supplementary Figure 2. Rac1 negatively regulates the number of TNT-connected cells.

a. Representative confocal images showing intercellular connections upon ectopic expression of Myc-Rac1 V12 (constitutively active form), Myc-Rac1 17N (dominant negative form), and their respective control, mCherry (red). Myc was detected by indirect immunofluorescence (red). Cells were observed by confocal microscopy. Scale bar = 10 µM. b) Quantification of TNT-connected cells upon ectopic expression of mCherry, Myc-Rac1 V12, and Myc-Rac1 17N. The ratio of TNT-forming transfected cells/number of transfected cells was evaluated. Data represents the mean (±SEM), normalized to control cells (mCherry transfected cells) arbitrarily set at 100%, of 3 independent experiments. *P < 0.05; **P < 0.01; ***P < 0.001; ns = not significant.

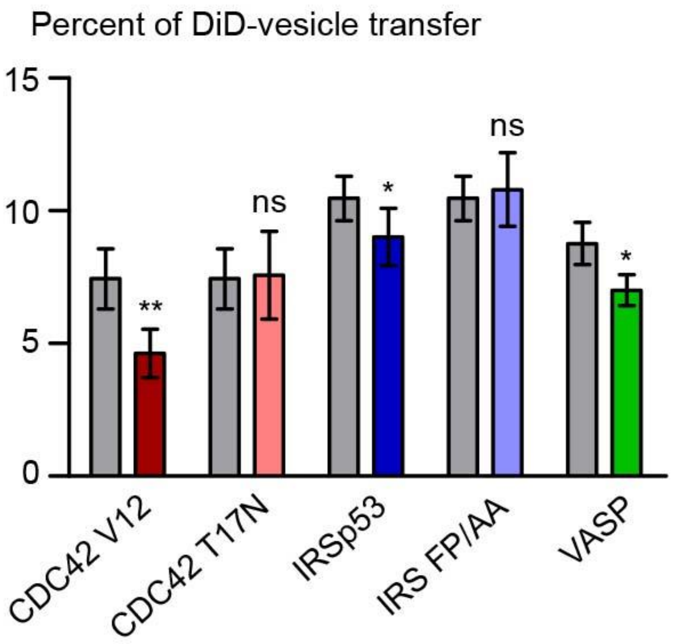

Supplementary Figure 3. CDC42, IRSp53, and VASP negatively regulate intercellular vesicle transfer. Quantification by flow cytometry of DiD-positive acceptor cells upon ectopic expression of GFP-CDC42 V12, GFP-CDC42 T17N, RFP-IRSp53, RFP-IRS FP/AA or GFP-VASP (colored bars) in the donor population, compared to respective control (i.e. GFP or RFP, grey bars). The percentage of DiD-positive acceptor cells in the total cell population was evaluated. Data represent the mean (±SEM) of at least 4 independent experiments. *P < 0.05; **P < 0.01; ***P < 0.001; ns = not significant.

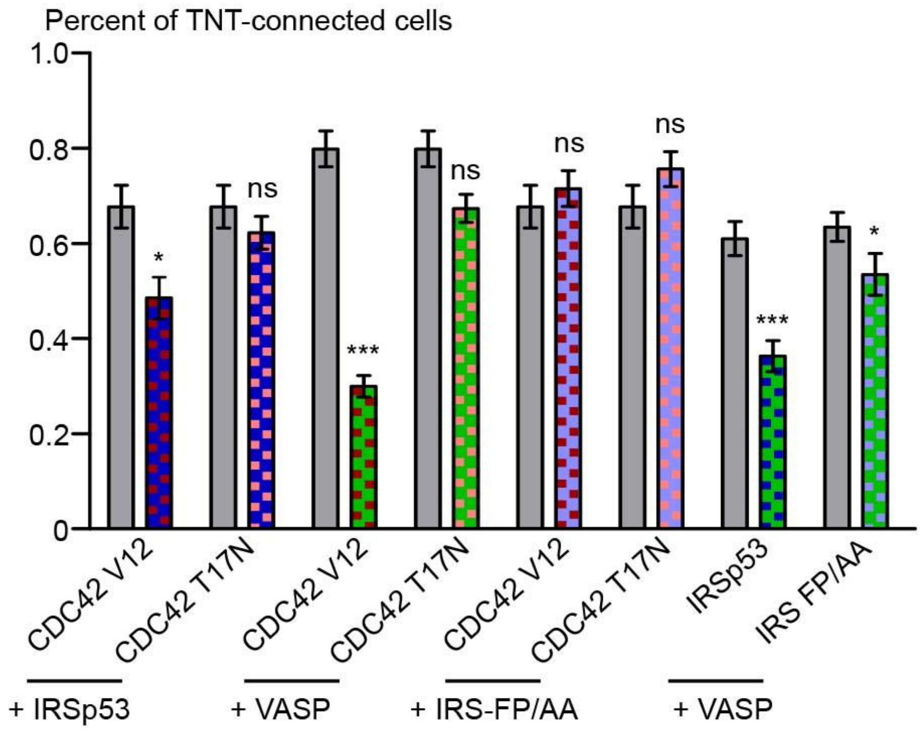

Supplementary Figure 4. CDC42, IRSp53, and VASP act as a network to negatively regulate the number of TNT-connected cells.

Quantification of TNT-connected cells upon co-transfection as indicated under the graph (colored bars) compared to respective controls (grey bars). Data represent the mean (±SEM) of at least 4 independent experiments. *P < 0.05; **P < 0.01; ***P < 0.001; ns = not significant.

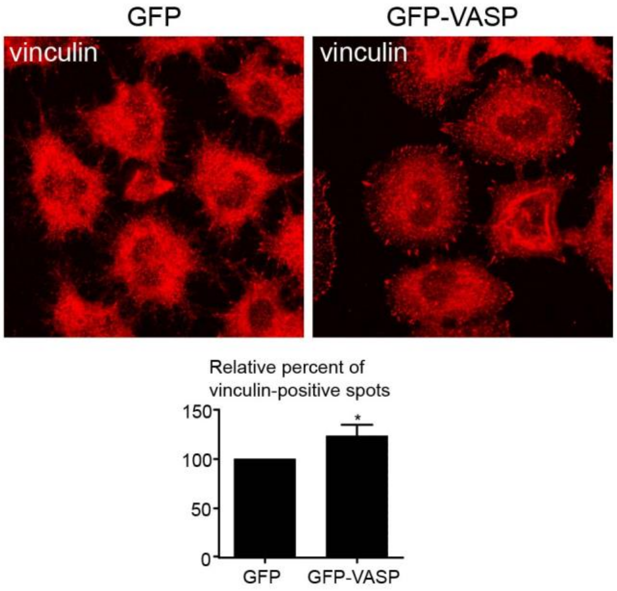

Supplementary Figure 5. Ectopic expression of VASP increases the number of vinculin-positive cellular protrusions

Cells were transiently transfected with GFP-VASP or GFP as a control. Cells were fixed 16 hrs post-plating and vinculin was detected by indirect immunofluorescence. The number of vinculin-positive peripheral focal adhesions was quantified using ICY. Data represent the mean (±SEM), normalized to control cells arbitrarily set at 100%, of at least 4 independent experiments. *P < 0.05.

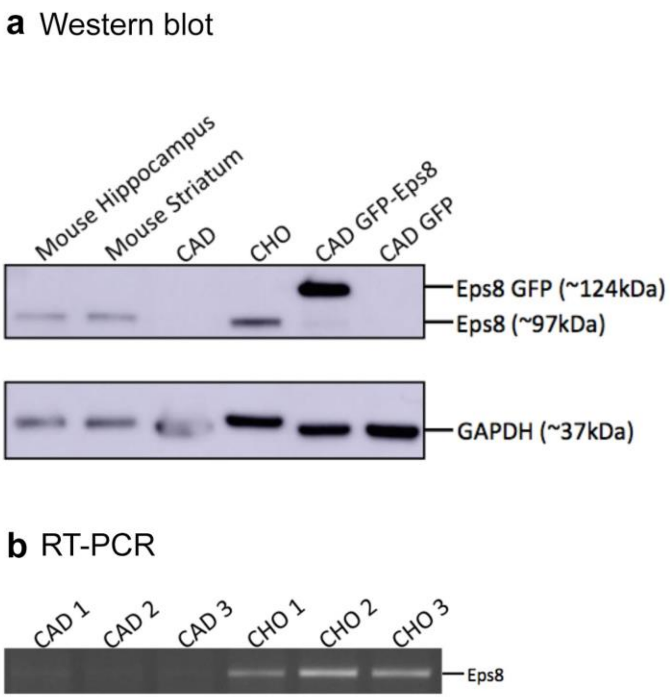

Supplementary Figure 6. Endogenous expression of Eps8 via western blotting and RT-PCR

a. Protein lysates from hippocampi and striata from mice at postnatal 21 days, CAD, CHO, CAD overexpressing GFP-Eps8, and CAD overexpressing GFP cells were immunoblotted for Eps8. 50 µg of protein per lane. b. RT-PCR analysis of Eps8 mRNA in CAD and CHO cells.

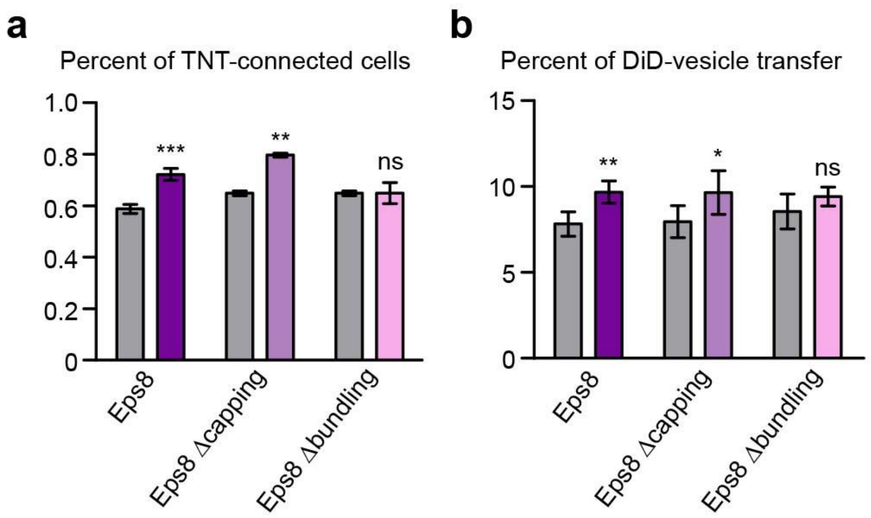

Supplementary Figure 7. Eps8 positively regulates TNT formation and intercellular vesicle transfer via its bundling activity.

a. Quantification of TNT-connected cells upon ectopic expression of GFP-Eps8, GFP-Eps8Δcapping or GFP-Eps8Δbundling (colored bars), compared to their respective control (grey bars). b. Quantification by flow cytometry of DiD-positive acceptor cells upon ectopic expression of GFP-Eps8, GFP-Eps8Δcapping or GFP-Eps8Δbundling (colored bars) in the donor population, compared to respective control (grey bars). Data represent the mean (±SEM) of at least 3 independent experiments. *P < 0.05; **P < 0.01; ***P < 0.001; ns = not signifiant.
